# Space-charge Effect on Electroresistance in Metal-Ferroelectric-Metal capacitors

**DOI:** 10.1038/srep18297

**Published:** 2015-12-16

**Authors:** Bo Bo Tian, Yang Liu, Liu Fang Chen, Jian Lu Wang, Shuo Sun, Hong Shen, Jing Lan Sun, Guo Liang Yuan, Stéphane Fusil, Vincent Garcia, Brahim Dkhil, Xiang Jian Meng, Jun Hao Chu

**Affiliations:** 1 National Laboratory for Infrared Physics, Shanghai Institute of Technical Physics, Chinese Academy of Sciences, Shanghai 200083, China; 2 University of Chinese Academy of Sciences, No.19A Yuquan Road, Beijing 100049, China; 3 Laboratoire Structures, Propriétés et Modélisation des Solides, CentraleSupélec, CNRS-UMR8580, Université Paris-Saclay, Châtenay-Malabry Cedex 92295, France; 4 School of Materials Science and Engineering, Nanjing University of Science and Technology, Nanjing 210094, China; 5 Unité Mixte de Physique, CNRS, Thales, Univ. Paris-Sud, Université Paris-Saclay, 91767, Palaiseau, France

## Abstract

Resistive switching through electroresistance (ER) effect in metal-ferroelectric-metal (MFM) capacitors has attracted increasing interest due to its potential applications as memories and logic devices. However, the detailed electronic mechanisms resulting in large ER when polarisation switching occurs in the ferroelectric barrier are still not well understood. Here, ER effect up to 1000% at room temperature is demonstrated in C-MOS compatible MFM nanocapacitors with a 8.8 nm-thick poly(vinylidene fluoride) (PVDF) homopolymer ferroelectric, which is very promising for silicon industry integration. Most remarkably, using theory developed for metal-semiconductor rectifying contacts, we derive an analytical expression for the variation of interfacial barrier heights due to space-charge effect that can interpret the observed ER response. We extend this space-charge model, related to the release of trapped charges by defects, to MFM structures made of ferroelectric oxides. This space-charge model provides a simple and straightforward tool to understand recent unusual reports. Finally, this work suggests that defect-engineering could be an original and efficient route for tuning the space-charge effect and thus the ER performances in future electronic devices.

Research on resistive switching in metal-ferroelectric-metal (MFM) capacitors has been intensified recently due to its potential applications in the next generation of non-volatile memories and logic devices^1^,^2^,^3^,^4^. The resistance in MFM structures can show large variations depending on the orientation of the ferroelectric polarisation. The polarisation in thin ferroelectric layers induces surface charges which in turn induce a depolarizing field that acts against the polarisation. In an MFM structure, the polarisation charges at the ferroelectric/electrode interface are partially screened by the electrons of the electrodes minimizing the effect of the depolarizing field. This results into an asymmetry in the barrier heights of the electronic band structure as sketched in Fig. 1a. Assuming that electrons dominate the conductance, the barrier height is lowered on the front side of the polarisation (where the positive bound charges are located) while it is increased on the back side. By reversing the polarisation state with an external electric field, the energy profile at MFM interfaces can thus be modulated. The electronic resistance through the MFM structure can be switched between two states allowing either a larger (ON) or smaller (OFF) flow of the current from one electrode to the other. This is the so-called electroresistance (ER) effect^5^,^6^,^7^,^8^. For a ferroelectric material sandwiched between two different metallic electrodes, the typical energy profiles due to the incomplete screening are depicted in Fig. 1a: the two polarization states (pointing to the left or to the right) correspond to different average barrier heights. This imperfect screening is also affected by interfacial effects which can be ascribed to defect-free layers having a different polarisation from that of the ferroelectric core and/or so-called dead-layers originating from non-switchable interface defects or pinned dipoles^9^,^10^,^11^. Yet, none of these aforementioned interfacial mechanisms do change drastically the profile asymmetry as the interfacial barrier height remains lower on the side to which the polarisation is pointing, i.e. the overall energy profile in Fig. 1a is conserved. Nevertheless, variations of barrier heights that drastically contrast with the expected energy scheme of Fig. 1a were reported very recently in La_0.7_Sr_0.3_MnO_3_ (LSMO)/BaTiO_3_/(Au or Cu) ferroelectric tunnel junctions (FTJs)^12^. The interfacial effect, which was considered phenomenologically to explain these results^12^, is based on density functional theory^12^,^13^,^14^ and corresponds to a profile asymmetry due to different atomic terminations. Such asymmetric interfacial effect exists even if the same electrode materials are used on both sides of the tunnel barrier, as shown by first-principles calculations^14^. Other recent works^15^,^16^ also reported unusual barrier heights and resistance state behaviours suggesting that the less common situation described above may occur much more often than expected. Here, by studying the ER effect in MFM structures based on 8.8-nm thick ferroelectric poly(vinylidene fluoride) [PVDF, -(CH_2_-CF_2_)_n_-], we reveal that unusual energy profiles at interfaces can be easily understood using a simple space-charge mechanism. Such interfacial effect competes with polarisation-based imperfect-screening giving rise to various energy profile situations. Similarly to basic semiconductors, charged defects like oxygen vacancies in oxides or charge-trapping defect sites in polymers may release their mobile/free charges at the interface and thus modify subsequently the energy profile. In addition, we show that the ER effect in such PVDF-based capacitors can reproducibly reach ~1000% ON/OFF ratio at room temperature. Moreover, these MFM structures are integrated onto silicon substrate and are thus C-MOS compatible, opening the route for future applications.

## Results

### Electroresistance in Au/PVDF/W nanocapacitors

Great efforts have been devoted to exploit the ER effect in MFM capacitors based on inorganic perovskite oxides as ferroelectric materials. Organic ferroelectrics are another interesting choice to develop highly flexible and low cost nonvolatile memories and logic devices^17^,^18^,^19^,^20^,^21^. Indeed, some polymers like poly(vinylidene fluoride) (PVDF) and its copolymers can retain a robust ferroelectricity (9 ~18 μC/cm^2^) even at the nanoscale^22^,^23^,^24^,^25^, with excellent chemical stability, switching characteristics and low production costs^26^,^27^,^28^,^29^,^30^. These features make organic MFM structures competitive alternatives and stimulate very active research. As a result, resistive switching depending on the orientation of the polarisation in organic MFM capacitors has indeed been observed with an ON/OFF ratio of about 10–100%^17^,^18^,^19^,^20^,^21^. Although experiments suggest that the ER in those organic polymers is associated with ferroelectric polarisation switching, the mechanism of current transport and the fundamental physics at the origin of the ER effect are still under debate and require further insights.

**Figure 1:**
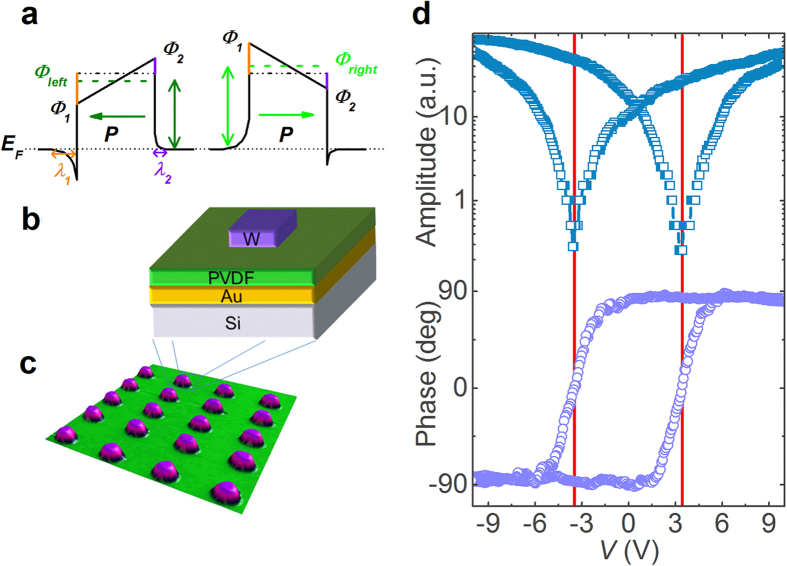
Ferroelectric switching in Au/PVDF/W nanocapacitors. (**a**) Schematic representation of the electron energy profiles across the ultrathin ferroelectric film: the potential profile with positive bound charge is lower than that with negative bound charge. The arrows denote the polarisation direction. *λ*_1_ and *λ*_2_ are the effective screening lengths of the left and right electrodes, respectively. 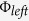 and 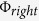 are the resulting average barrier heights for polarisation pointing left and right, respectively. (**b**) Sketch of the Au/PVDF/W nanocapacitor on Si substrate. (**c**) AFM topography image of 20 typical nanocapacitors. (**d**) Out-of-plane PFM amplitude (top panel) and phase (bottom panel) hysteresis loops of an Au/PVDF/W nanocapacitor. The red lines indicate the coercive voltages.

Pure PVDF ultrathin films were fabricated by the Langmuir-Blodgett (LB) deposition technique. Four layers of ferroelectric PVDF with a total thickness d of ~8.8 nm (ref. 23) were transferred onto Au-coated Si substrates. A tungsten (W) top electrode was locally deposited onto the films using electron-beam-induced deposition to finalize the nanoscale MFM capacitors. A sketch of the corresponding structure is presented in Fig. 1b,c. Figure 1b depicts the device structure while Fig. 1c shows an atomic force microscope (AFM) topography image of 20 of these nanocapacitors. The lateral diameter of each capacitor is about 360 nm. The ferroelectric character of these ultrathin films was previously demonstrated by standard polarisation vs. field loops down to 5 layers^23^. It is worth recalling that while homopolymer PVDF films were believed to be obtained in their non-polar *α*-phase, it was recently shown that ultrathin PVDF films fabricated by LB deposition technique crystallize in their polar *β*-phase, as confirmed by infrared spectra and X-ray diffraction^24^. Moreover the *β*- to *α*-phase transition in LB PVDF films was also demonstrated by dielectric measurements^25^. Furthermore, compared to LB P(VDF-TrFE) copolymer films, the LB PVDF films are also found to display better ferroelectric properties (higher polarisation, higher breakdown electric field^23^, and endurance over more than 10^5^ polarisation switching cycles^26^). To confirm the ferroelectric character of PVDF ultrathin films within the nanocapacitors, we used piezoresponse force microscopy (PFM) to probe the ferroelectric response of PVDF through the top electrode of W. The out-of-plane PFM amplitude and phase hysteretic loops show the typical signal expected for ferroelectric materials (Fig. 1d), from which we derived the coercive voltage of PVDF as V_*C*_ ~ −3.6 V, +3.5 V (see the red vertical lines).

**Figure 2:**
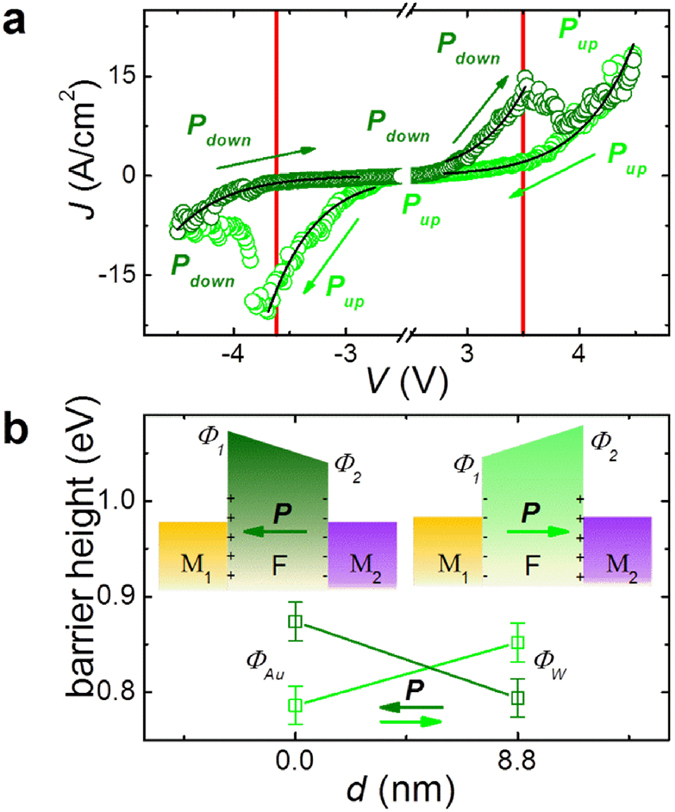
ER effect in a Si/Au/PVDF/W nanocapacitor. (**a**) Current density versus voltage (*J-V*) curves of a Au/PVDF/W nanocapacitor. Solid lines are fits using the thermionic injection model. The arrows show the path of the current. The red lines indicate the coercive voltages from polarisation switching in Fig. 1d. (**b**) Barrier heights statistically derived from the TI fittings. The insets show schematic representation of energy potential profiles across the ultrathin ferroelectric PVDF films and the arrows denote the directions of the polarisation.

To measure the electronic transport properties of the capacitors, an electrical contact on the top electrode W pad was made using a conductive-tip AFM (C-AFM)^19^. The voltage was applied to the bottom Au electrode while the top W electrode was grounded. As indicated in Fig. 2a, the current shows a dramatic decrease both at the positive coercive voltage (when sweeping the voltage from −4.5 V to +4.5 V, dark green points) and the negative coercive voltage (when sweeping the voltage from +4.5 V to −4.5 V, light green points), while no current is detected in the low voltage region because of the instrument detection limit. The resistive switching occurs at −3.6 ± 0.2 V and +3.5 ± 0.2 V depending on the device. It appears that the resistive switching occurring in these PVDF-based MFM capacitors is indeed associated with the polarisation reversal (red lines in Fig. 2a). It is tempting to claim that the dramatic change in the resistance at the coercive voltage is solely attributed to the displacement current due to polarisation switching^31^. However, it can be definitely ruled out here since the current density (*J*) is of the order of 1 A/cm^2^ which corresponds to a charge density of ~1 C/cm^2^ by integrating it with the current duration time of ~1s. Such charge density represents an equivalent polarisation which is about 6 orders of magnitude larger than that commonly reported in PVDF thin films (~1 μC/cm^2^, ref. 22 and 23). In addition, the current shows almost no decay when doubling the time period of the current density versus voltage (*J*-*V*) measurement (Supplementary Fig. S1).

The ER effect is reproducible and stable (Supplementary Fig. S2a). The resistance-area product in these Au/PVDF/W nanocapacitors is 0.1~1 Ω·cm^2^ for the ON state (Supplementary Fig. S2b) and the ER (OFF/ON ratio) reaches values over 1000% at room temperature (Supplementary Fig. S2a). These characteristics for the PVDF nanocapacitors suggest potential applications as electronic devices. Since the fabrication process of PVDF-based MFM capacitors on silicon substrates is simple and reliable, all aforementioned characteristics make them very promising as efficient, low cost and C-MOS compatible (not mentioning the compatibility of PVDF with flexible substrates) electronic elements.

To get insights into the observed ER effect, we need to identify the electronic transport mechanism occurring through the Au/PVDF/W nanocapacitors. Note that the ferroelectric films we used are relatively thick (~8.8 nm), so that thermionic injection (TI) rather than direct tunnelling is thus expected to play a dominant role in the electronic transport properties at room temperature^5^,^32^. The Fowler-Nordheim tunnelling (FNT) mechanism has also been considered but it cannot explain the current vs. voltage obtained experimentally (see Supplementary Information). According to TI, the corresponding electric field (*E*) dependence of the current density (*J*_*TI*_) is determined by^33^,^34^,^35^


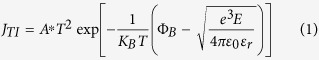


where 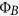, 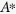, 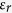, *T* are the potential barrier, the effective Richardson’s constant, the permittivity of the ferroelectric responsible for image force lowering, and the temperature, respectively. The effective electric field (*E*) across the ferroelectric films is a superposition of the applied field 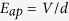, the depolarizing field and the field due to the band alignment (see Supplementary Information)^5^,^8^. Similarly to most oxide ferroelectrics, the barrier heights at the PVDF/metal interfaces are lower at the conduction band than at the valence band. Therefore, electrons can be considered as the mobile carriers^36^,^37^. Figure 2a shows the fitting results (black lines) of the experimental *J*-*V* data using Equation (1) (We fit our data using the same method as in reference 5 and in the fits the parameters 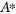 = 1×10^6^ Am^−2^K^−2^, *S* = 1×10^−9^ cm^−2^, *d* = 8.8 nm, 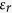 = 3, *T* = 295 K were fixed). TI process dominates the electronic transport behavior in this 8.8-nm PVDF-based MFM capacitor, as seen by the very good fitting agreement. To obtain a statistical evaluation on the barrier heights variations, the fitting procedure was repeated for five different nanocapacitors and using on average five *J*-*V* cycles for each junction. The corresponding results are summarised in Fig. 2b (bottom).

It is shown that the barrier heights of PVDF/W and Au/PVDF interfaces vary from 0.78 eV to 0.83 eV and from 0.85 eV to 0.77 eV, respectively when the polarisation is switched from the left to the right state. Interestingly, this situation corresponds to an increase of barrier height at both ferroelectric/metal interfaces where the polarisation is pointing to (as sketched in Fig. 2b, top). This behaviour, similar to the one recently reported for LSMO/BTO/(Au, Cu)^12^, is in contrast to the typically expected energy profile with imperfect-screening, observed for instance in epitaxial Pb(Zr_0.2_Ti_0.8_)O_3_ films^5^.

### Space-charge mechanism as interface effect in Au/PVDF/W capacitors

**Figure 3:**
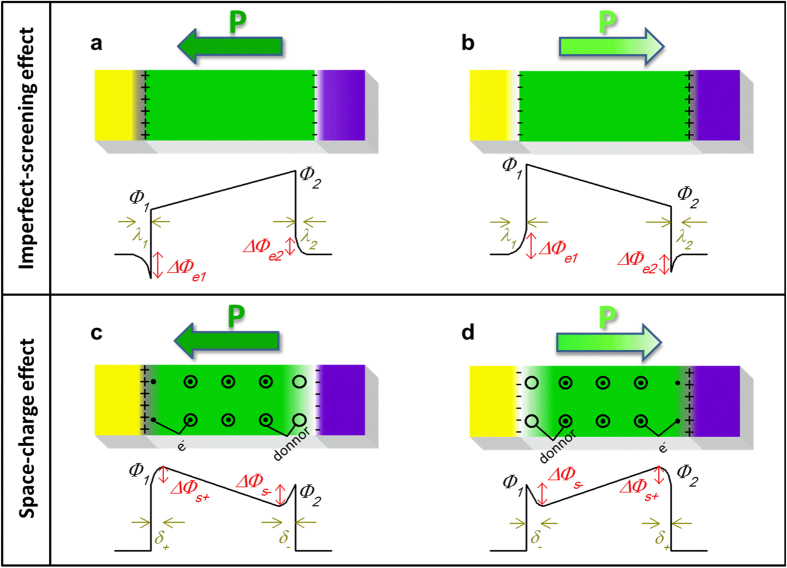
The effect of polarisation bound charges on energy potential profiles. Schematic representation of the energy potential profiles of the MFM structure considering only the electrode-imperfect-screening effect when polarisation points to left side (**a**) and right side (**b**), or only the space-charge effect when polarisation points to left side (**c**) and right side (**d**). Insets are sketches of the MFM structure. A depletion layer with positive charges is formed near the negative bound charges.

To understand the foregoing discrepancy, we make further analysis on the energy potential by treating the ferroelectric as an n-type non-degenerated semiconductor and assuming thermodynamic equilibrium. Note that using a similar treatment with p-type semiconductor would give the same result. For the case of fully depleted ferroelectric thin films (Fig. 3a), imperfect screening for polarisation bound charges in the electrodes leads to a drop of 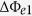 of the barrier height on the positive bound charges side and a rise of 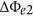 on the negative bound charges side^7^,^38^. Recent ab-initio works have shown that the quality of the screening strongly depends on the interface bonding structure (chemical interaction between the two materials)^39^ (see Supplementary Information). However, considering that the PVDF films are attached to electrodes by Van der Waals force which is much weaker than the interface chemical interaction, the screening effect here is estimated using the classical Thomas-Fermi theory. Then the change of the interfacial barrier height is determined as^7^,^8^,^12^:


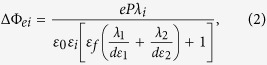


where 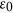 is the permittivity of free space, 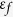 is the relative permittivity of the ferroelectric layer, 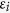 is the relative permittivity, and 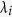 is the Thomas-Fermi screening length in of each electrode *i* (*i* = 1 or 2)^8^,^12^.

If there are mobile charges in the ferroelectric layer, they will remarkably modify the energy configuration. For example, treating the ferroelectric as an n-doped semiconductor, the bound charges are compensated by the formation of thin layers of charges with opposite sign in the ferroelectric layer as illustrated in Fig. 3c,d. Here we temporarily ignore the variation of barrier height due to the imperfect screening from electrodes as displayed in Fig. 3a,b. Then, the polarisation leads to an increase of the barrier height by 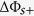 on the positive bound charges side and a decrease by 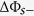 on the negative bound charges side^40^,^41^. By combining both imperfect-screening (Fig. 3a) and space-charge effects (Fig. 3c), several situations can be obtained depending on the relative amplitudes of 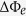 and 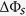. In our case, 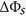 is considered to be bigger than 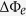 and thus the contribution of space charges dominates the electrostatic imperfect-screening effect. It is worth mentioning that the top tungsten electrode in our MFM structures was deposited on the PVDF layer by electron-beam-induced method, so that high energy electrons might induce numerous defects^42^ even if the electron irradiation did not destroy the ferroelectricity of our PVDF films, as confirmed by the PFM hysteresis loops. Putative defects may serve as energy traps for mobile charges that can be released at the interfaces to compensate the bound charges. In this very simplistic model, no assumption on the polarisation value at the interfaces (dead-layer^9^,^11^ nor defect-free layer with different polarisation amplitude^43^ nor different polarisation because of different termination^44^) is made, and the charge-trapping defects can be either located at the interfaces or in the whole volume of the ferroelectric layer.

In order to describe quantitatively the effect of space charges on the variation of the barrier height, we used the theory developed for metal-semiconductor rectifying contacts^45^. The Poisson equation for the structure presented in Fig. 3c,d is expressed as:


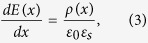


where 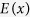 is the electric field, 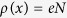 is the charge density and 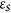 is the relative permittivity in the space-charge layer. The boundary conditions for the electric field are: 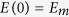 (at the interface, the electric field is maximum); 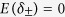 (the electric field in the neutral volume is zero)^45^. 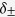 is the width of the compensating layer near the positive (+) or negative (−) bound charge sides and can be estimated as: 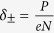. For an n-type film, 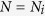 is the effective density of states at the interface of the positive bound polarisation charges side, while 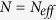 is the effective charge density in the depleted region at the negative bound charges side (taking into consideration all the charges, including the trapped ones). *P* is the polarisation of the ferroelectric layer. The energy potential bending due to the polarisation can be obtained by integrating the electric field from the interface over the width of the compensating layer:


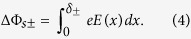


Combining Eq. (3) and Eq. (4), we can obtain: 
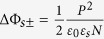
.

Both the electrode-imperfect-screening effect and the space-charge effect can compete in practical situations. Given that in our case *λ*_*i*_*, δ*_*i* ±_ ≪ *d*, the variation of barrier height in the interface can be approximately expressed as: 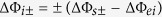, where the “±” stands for the sign of bound charges due to polarisation. It is found that 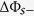 = 0.32 eV is much larger than 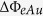 = 0.10 eV, 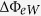 = 0.078 eV and 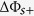 = 0.032 eV by using the following typical parameters^23^,^46^,^47^,^48^,^49^: *P* = 3 μC/cm^2^, 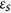 = 5, 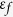 = 6, 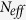 = 2 ×10^20^ cm^−3^, *N*_*i*_ = 2 × 10^21^ cm^−3^, 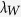 = 0.048 nm, 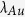 = 0.06 nm and 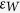 = 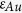 = 2.

As sketched in Fig. 3c,d, there is a depletion region (with the thickness *δ*- = 0.94 nm) near the negative bound charges. It is this significant drop of potential at negative bound charges side 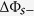 = 0.32 eV that leads to the phenomenon observed in this experiment: because of the space charges, the barrier height is much more altered on the negative polarisation bound charges side which results on a higher barrier height on the side where the polarisation is pointing to. Here, the space-charge mechanism outstrips the classical imperfect-screening process.

### Evaluation of space-charge effects in oxide-based capacitors

Our space-charge model succeeds to interpret the unusual data obtained in the studied PVDF-based MFM structure. Such space-charge effect remains also valid whatever the nature of the ferroelectric (organic or inorganic, with n- or p-type doping). Similar and unusual variations of barrier heights were for example reported in inorganic BaTiO_3_-based ferroelectric junctions^12^. For inorganic oxide films, an excess of oxygen vacancies (n-doping defects) resulting from the poor-oxygen atmosphere in fabrication process or surface oxygen exchange close to the top surface may happen^41^,^50^, and the defect density can be as large as ~10^21^–10^22^ charges/cm^3^ (ref. 51, 52, 53). Based on the results on PVDF-structures, one may wonder if such oxygen vacancies can play the role of space charges and can thus be at the origin of the unusual energy profiles observed experimentally. This assumption will be tested by the following analysis in the same framework as already described for PVDF-based devices.

**Figure 4:**
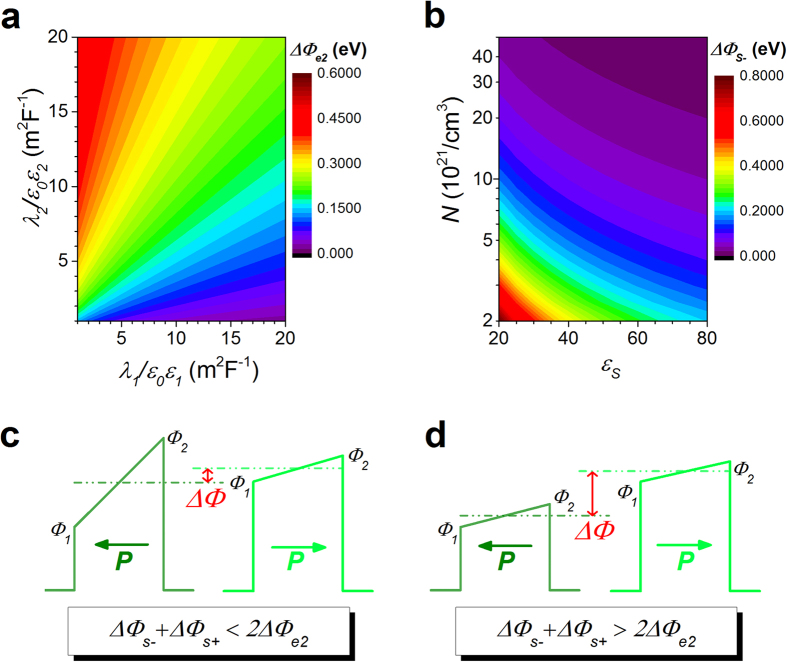
Competition between electrode-imperfect-screening effect and space-charge effect. The quantitative variation of barrier height by electrode-imperfect-screening effect (**a**) and space-charge effect (**b**) taking the following parameters: 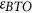 = 200, *P* = 30 *μ*C/cm^2^ and *d* = 3 nm. Schematic energy potential profiles of the MFM structure in the two polarisation states, considering that some defect charges exist near interface 2, for two different cases: 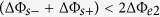 (**c**), and 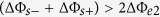 (**d**).

Figure 4a,b shows the quantitative variation of barrier height by electrode-imperfect-screening and space-charge effect, when considering an MFM structure made of BaTiO_3_ (BTO) sandwiched between two electrodes M_1_ (LaSrMnO_3_ or SrRuO_3_) and M_2_ (Au or Cu). 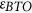 = 200, *P* = 30 *μ*C/cm^2^ and *d* = 3 nm are taken in the calculations to fit this inorganic structure^12^. Note that 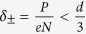 is taken into consideration to meet the interface approximation (the thickness of the interface should be far smaller than that of the films). From the calculations, it appears that depending on the defect density near the interface the variation of barrier height due to the space-charge effect (Fig. 4b) can be larger, equal or smaller than that arising from the electrode-imperfect-screening effect (Fig. 4a). The LSMO/BTO/(Au, Cu) junctions (LSMO is the bottom electrode) are analysed on the basis of these calculations (Fig. 4a,b). The unusual variations of barrier heights reported in ref. 12 are well fitted using the model taking space-charge effect into account (See Supplementary Information).

Using this space-charge model, one may reconcile the body of experiments in FTJs with different electrodes. In ref. 12, the interface capacitance (*C*) inequality, 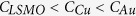, is obtained based on experimental measurements and the imperfect screening model. The change in the mean barrier height after polarisation reversal (by switching the polarisation from left to right) is:


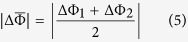


where 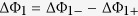 and 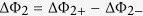 stand for the variation of barrier height at interface 1 (bottom interface) and interface 2 (top interface) respectively. The imperfect screening model (eq. (2)) gives the solution: 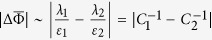. Since 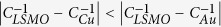 and the tunnelling ER is mostly governed by 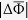, the imperfect screening model leads to an increase in the tunnelling ER ratio after the replacement of the Cu TE by the Au one. However, this is opposite to the experimental observations^12^. This contradiction is solved taking into account the space-charge effect. Based on the new model, the smaller interface capacitance *C*_*Cu*_ than *C*_*Au*_ can be realized with a smaller permittivity or a bigger thickness (corresponding to a lower charge density) of the space-charge layer for the BTO/Cu interface. According to Fig. 4b and 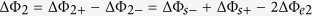, this will lead to a larger change of barrier height of BTO/Cu interface than that of BTO/Au interface. Indeed, this is confirmed by the experimental observations^12^. Furthermore, the higher tunnelling ER ratio in LSMO/BTO/Cu junction implies a bigger variation of mean barrier height. Assuming that similar space-charge effect occurs on interfaces 1 and 2, the change of mean barrier height is not affected by the space-charge effect, and one can obtain: 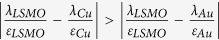. A potential solution of this inequality, 
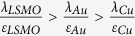
, accords well with the Thomas-Fermi theory where 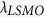 = 0.2~1.9 nm, 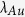 = 0.06 nm, 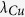 = 0.055 nm, 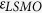 = 8, 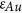 = 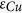 = 2, respectively^8^,^47^,^49^,^54^,^55^.

The space-charge effect can then be used to design or improve devices. For example, a dedicated surface treatment or spontaneous oxygen exchange close to the top surface^41^,^50^ can form a single charged defects layer only at the interface 2 while almost no charged defects are present at interface 1. Therefore both electrode-imperfect-screening effect and space-charge effect can compete when the polarisation points to interface 1, while space-charge effect is absent when the polarisation points to interface 2. The change of barrier height at the interface 1 after the polarisation reversal is: 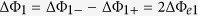, while at the interface 2 it is: 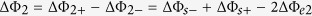. Strikingly, space-charge effects here result in a strong boost of 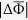 as compared to only imperfect-screening effects. That is: 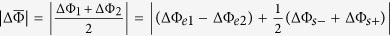, where 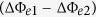 is attributed to the imperfect-screening effect and 
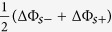
 is the contribution from space-charge effect. Note that, for the usual inorganic FTJs, the top noble metal electrodes (such as Au or Pt) generally have better electronic screening than bottom oxide electrodes (such as LaSrMnO_3_ or SrRuO_3_)^39^,^55^, then both 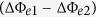 and 
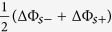
 are positive. The space-charge contribution improves the average barrier shift between the two polarisation orientations, which will in turn result in a larger ER effect as already anticipated^7^,^8^.

The situation schematized in Fig. 4c gives similar results to that depicted in Fig. 1a: the imperfect-screening effect is dominating the space-charge one. In that case, either a very high density of mobile charges is present resulting in a very negligible role of the space-charge effect (Fig. 4b) or the film is insulating (or entirely depleted) and the space-charge effect plays no role in the MFM capacitor. The former situation seems unlikely to happen in real situations because high density of mobile charges will lead to the decrease or disappearance of the barrier layer through the increase of the Femi energy (*E*_*F*_) level in the film. On the contrary, for high-quality ultrathin ferroelectric films, i.e. when, for example, the mobile charge density is lower than 5×10^20^ charges/cm^3^, the width of the depletion layer (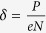) for a polarisation of *P* = 30 *μ*C/cm^2^ can be larger than 3.75 nm. The ultrathin ferroelectric film is then treated as an insulator (entirely depleted) as it is the case in several reports^19^,^56^,^57^.

## Discussion

A simple model based on space-charge effect is proposed here to explain the variations of the barrier height observed in our PVDF-based MFM capacitors as well as in some recent experimental reports on inorganic oxide-based capacitors. There is then no systematic need to invoke any other interfacial effects like dead-layers, polarisation-modified layers and/or different chemical terminations at the interface. Actually, the space-charge effect related to the semiconducting character of the ferroelectric film may compete with the imperfect screening of polarisation charges from the electrodes. This space-charge mechanism can be considered as an interfacial effect and treated electrostatically when mobile charges trapped by defect sites are released in the ferroelectric film.

Finally, the ER in MFM structures may be engineered and improved by controlling the space-charge effect through the defects in the ferroelectric film. This new defect-engineering approach opens a promising route for tuning the resistive switching in ferroelectric-based capacitors.

## Methods

### Sample Preparation

The PVDF homopolymer used in this work was prepared by a horizontal technology LB method. Bulk PVDF (with molecular weights of 180275, purchased from Sigma-Aldrich) was dissolved in dimethyl sulphoxide with a concentration of 0.01% wt. The polymer solvent was dispersed on the surface of deionized water with a resistivity of 18.2 MΩ • cm in a KSV LB trough (Nima 611). The bottom gold electrode was sputtered on the Si substrate. The PVDF layers were transferred from the water-air interface on gold-coated Si substrate with a surface pressure maintained at 5 mNm^−1^ at 25 °C in atmospheric environment. The tungsten top electrode was deposited onto the films through electron-beam-induced deposition method to form the gold/PVDF/tungsten capacitor structure.

### Measurements

PFM measurements were performed at room temperature using a commercial AFM (Bruker multimode 8) with a PFM mode. *I-V* curves measurements were performed by applying quasi-static voltage sweeps (measurement time period: T = 10 s and 20 s) to the bottom electrode, while the C-AFM tip connecting top electrode was grounded. We used commercial Pt-coated Si tips for the PFM and C-AFM experiments.

## Additional Information

**How to cite this article**: Tian, B.B. *et al*. Space-charge Effect on Electroresistance in Metal-Ferroelectric-Metal capacitors. *Sci. Rep*. **5**, 18297; doi: 10.1038/srep18297 (2015).

## Supplementary Material

Supplementary Information
