# Supplementary material for: Space-charge Effect on Electroresistance in Metal-Ferroelectric-Metal capacitors
Source: Sci Rep. 2015 Dec 16;5:18297. doi: 10.1038/srep18297 (PMC4680931; doi:10.1038/srep18297)
Supplement: Supplementary Information [file srep18297-s1.doc]

**Supplementary Information for**

**Space-charge Effect on Electroresistance in Metal-Ferroelectric-Metal capacitors**

Bobo Tian^1,2,3*^, Yang Liu^3^, Liufang Chen^4^, Jianlu Wang^1,2^, Shuo Sun^1,2^, Hong Shen^1,2*^, Jinglan Sun^1,2^, Guoliang Yuan^4^, Stéphane Fusil^5^, Vincent Garcia^5^, Brahim Dkhil^3*^, Xiangjian Meng^1,2^ and Junhao Chu^1,2^

^1^National Laboratory for Infrared Physics, Shanghai Institute of Technical Physics, Chinese Academy of Sciences, Shanghai 200083, China. ^2^University of Chinese Academy of Sciences, No.19A Yuquan Road, Beijing 100049, China. ^3^Laboratoire Structures, Propriétés et Modélisation des Solides, CentraleSupélec, CNRS-UMR8580, Université Paris-Saclay, Châtenay-Malabry Cedex 92295, France. ^4^School of Materials Science and Engineering, Nanjing University of Science and Technology, Nanjing 210094, China. ^5^ Unité Mixte de Physique, CNRS, Thales, Univ. Paris-Sud, Université Paris-Saclay, 91767, Palaiseau, France.

*^*^*Correspondence and requests for materials should be addressed to brahim.dkhil@centralesupelec.fr, tianbobo123@qq.com, and hongshen@mail.sitp.ac.cn.

**Contents**

**1, Current density and voltage (*J-V*) curves**

**2, Electroresistance characteristics**

**a) Resistance-area product (left) and ER ratio (right).**

**b) Resistance-area products at -3 V for n continuous *J-V* curve cycles.**

**3, Transport mechanisms**

**4, Possible space-charge effect to explain the unusual variation of the barrier height for LSMO/BTO/Au and LSMO/BTO/Cu junctions**

**1, current density and voltage (*J-V*) curves**

To measure the electronic transport properties, an electrical contact on the top electrode W pad was made using a conductive-tip AFM (C-AFM). The voltage was applied to the bottom Au electrode while the top W electrode was grounded. As already discussed in the main text, the resistive switching occurring around the coercive voltages is not attributed to solely the displacement current due to the polarization switching because the charge density from the *J-V* curves represents an equivalent polarization which is about 6 orders of magnitude larger than that commonly reported in PVDF thin films (see main text). It is believed that real charge leakage dominates in those Si/Au/PVDF/W nanocapacitors. To further confirm our point, the *J-V* measurement was performed under measurement period of 10 seconds and 20 seconds respectively. As shown in Fig. S1, the current density shows almost no decay when doubling the time period of the *J-V* measurement, which indicates that the resistive switching here is attributed to leakage rather than displacement current.


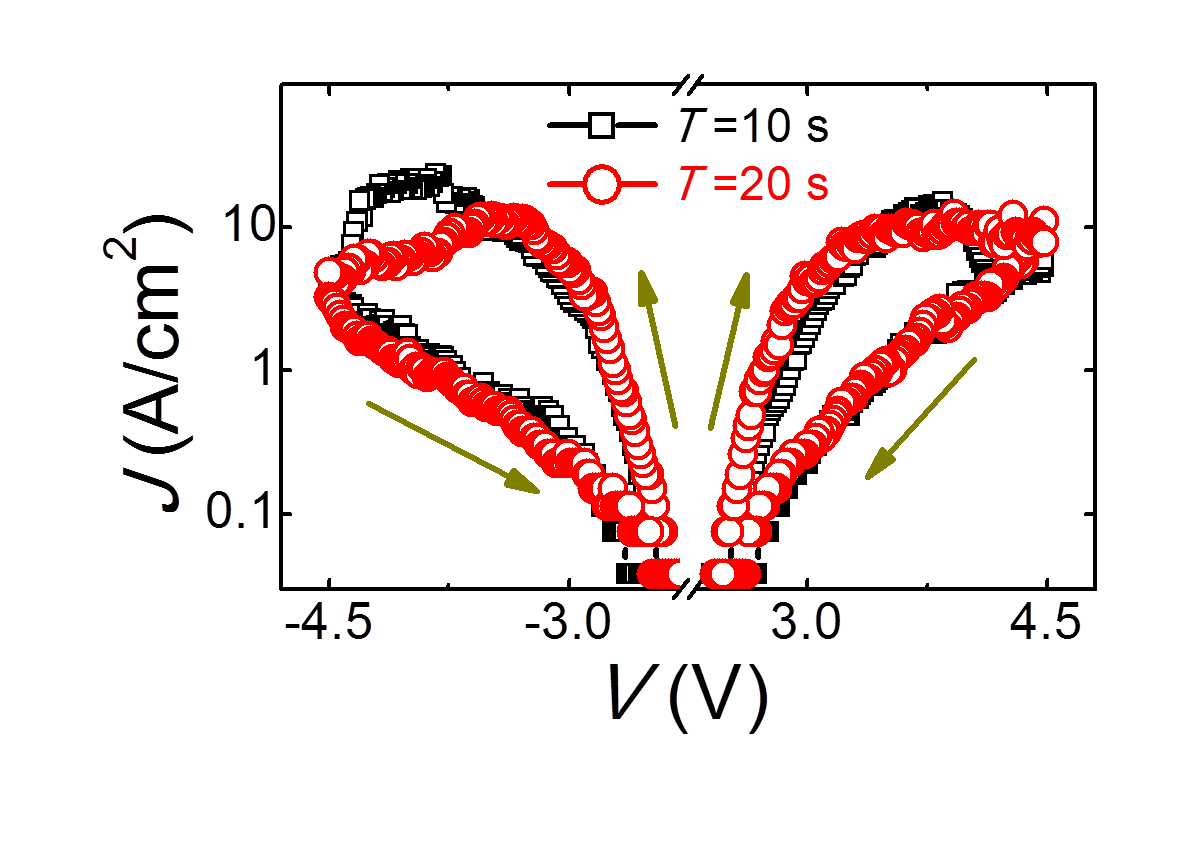


**Figure S1 | J-*V* curves.** *J-V* curves for the same nanocapacitor using measurement period of 10 seconds and 20 seconds. The arrow shows the path of the current.

**2, Electroresistance (ER) characteristics**

As shown in Fig. S2a, the resistance state for the same polarization orientation can change between the positive and negative voltage range. The small resistance-area product *R$\times$S* (~10 Ω·cm^2^ for OFF states) and large ER (ON/OFF ratio > 1000%) meet the basic requirements for potential electronic devices. The resistance-area product *R$\times$S* at -3 V for ten continuous *I-V* measurement cycles is presented in Fig. S2b and confirms that the ferroelectric ER here is reproducible and stable. Since the fabrication process of PVDF-based MFM capacitors on silicon substrates is simple and reliable, all aforementioned characteristics make them very promising as efficient, low cost and C-MOS compatible (not mentioning PVDF flexibility) electronic elements.


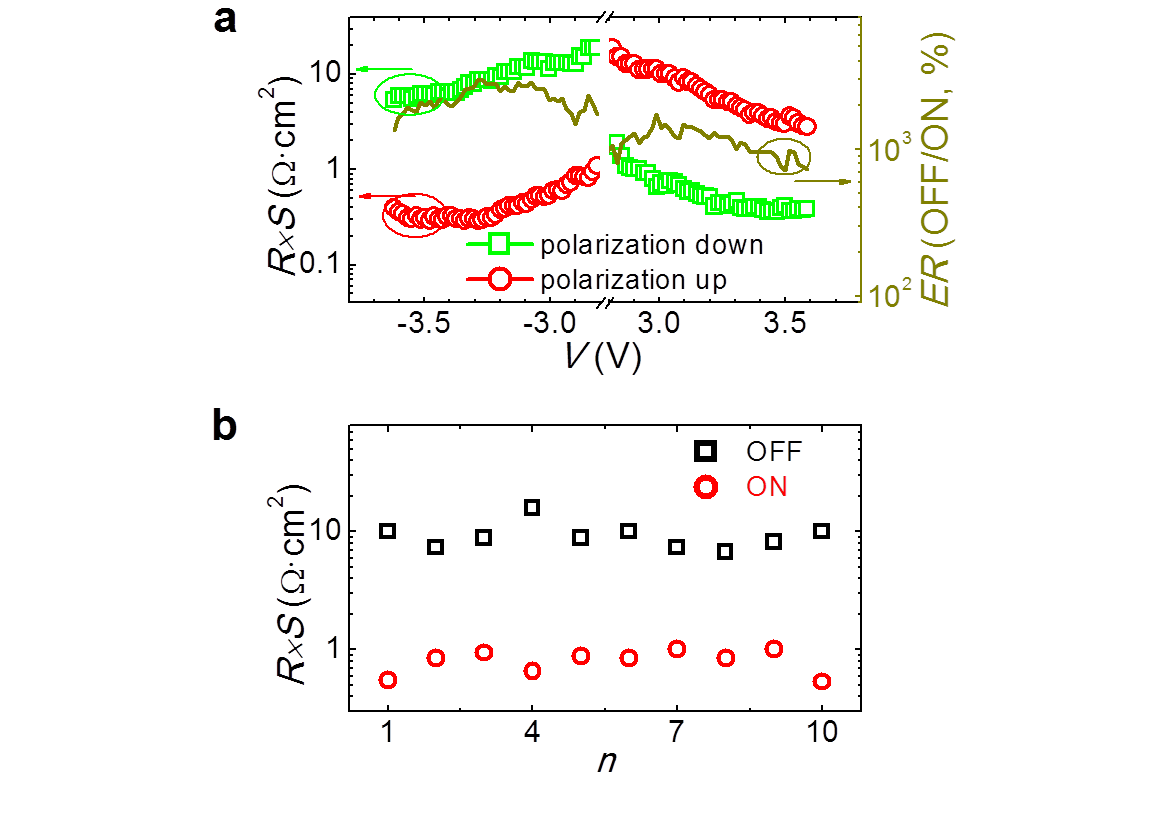


**Figure S2 | Electroresistance characteristics.** (a) Resistance-area product *R$\times$S* (left) and ER ratio (right). (b) Resistance-area product at -3 V for n continuous *J*-*V* curve cycles.

**3, Transport mechanisms**

In agreement with previous works^1^, direct tunneling mechanism can be neglected as the thickness of our ferroelectric films i.e. 8.8nm is too thick compared to the critical thickness which is ~3-5nm allowing electronic transport through thermionic injection rather than direct tunneling

Thermionic injection occurs when carriers overcome the potential barrier by thermal energy.^2,3^ Suppose a positive voltage is applied to metal 2, this current can be described by^1,3^

$I_{TI}=SA^{*}T^{2}\exp\left[-\frac{1}{K_{B}T}\left(\Phi_{1}-\sqrt{\frac{e^{3}E}{4\pi\varepsilon_{0}\varepsilon_{r}}}\right)\right]$ (1)

where *Ф*_1_, *A*^*^, *K*_B_, 𝜀_r_, *T* are the potential barrier height for interface 1, the effective Richardson’s constant, the boltzmann's constant, the permittivity of the ferroelectric responsible for image force lowering, and the temperature, respectively. The electric field *E* is the field responsible for band tilting, i.e., a superposition of the applied field *E*_ap_ = *V*/*d*, the depolarizing field (due to imperfect screening of polarization charges), and the field due to band alignment *E*_band_ = $\frac{\Phi_{1ori}-\Phi_{2ori}}{ed}$ (*Φ*_1ori_ and *Φ*_2ori_ are original barrier heights without being modified by polarization). The superposition of the depolarizing field and the field due to band alignment gives the final *Φ*_1_ and *Φ*_2_ at V=0. Then the electric field *E* for band tilting is: $E=\frac{E_{ap}+(\frac{\Phi_{1}}{e}-\frac{\Phi_{2}}{e})}{d}$.

Let us now consider that FNT may compete with TI under high bias voltage. FNT is flowing across a triangular-shaped potential barrier, which is formed by applying an electrical field *E* to a rectangular or trapezoidal barrier.[^4^](#_ENREF_9) Suppose a positive voltage is applied to metal 2, the current is given by[^4^](#_ENREF_9)

$I_{FNT}=S\frac{e^{3}}{8\pi h\Phi_{1}}E^{2}\exp\left[-\frac{8\pi\sqrt{2m^{*}}\Phi_{1}^{3/2}}{3heE}\right]$ (2)

where *E*, ℎ, *m** are field responsible for band tilting (the same as in equation (1)), Planck’s constant, and effective carrier mass, respectively.

In our case, the currents were therefore fitted using eq. (1) and eq. (2), respectively. The parameters used for the fitting are the same as in the main manuscript and *m*^*^ = m_0_ is used in eq. (2). As shown in Fig. S3, the TI (red lines) mechanism gives a much better agreement with the experimental data than the FNT (black lines) mechanism.


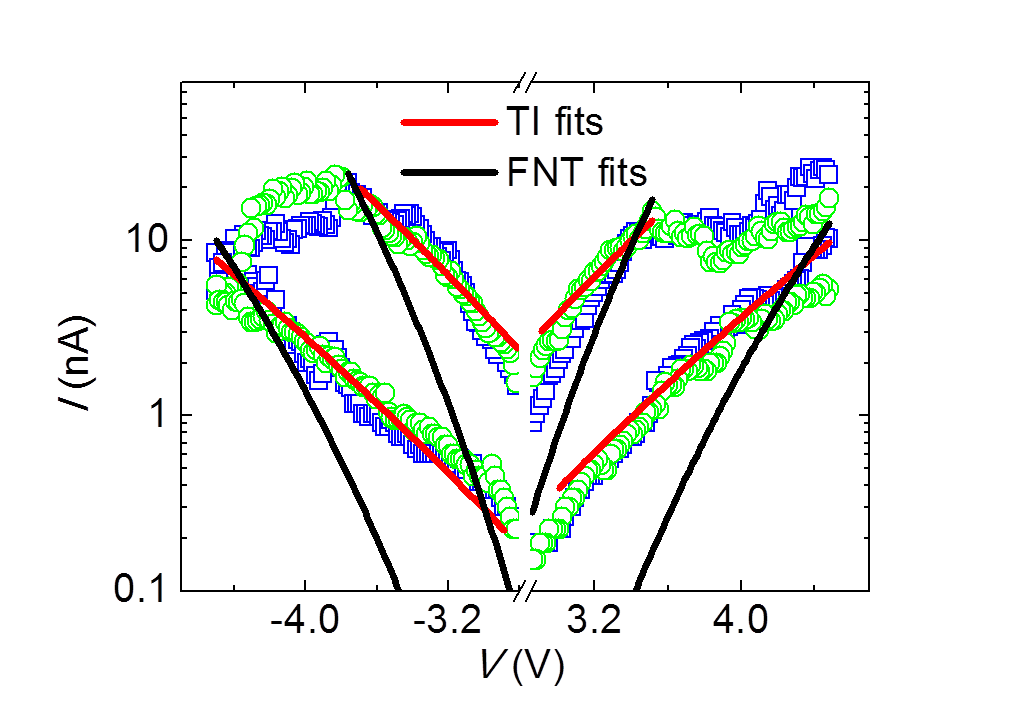


**Figure S3 | Transport mechanisms.** *I*-*V* curves of an Au/PVDF/W nanocapacitor (open symbols, two sets of measurements). Red lines and black lines are fits to those currents using TI formula and FNT formula, respectively.

It is worth mentioning that while excluded because of a worst agreement with the experiments, FNT gives similar variation of the barrier heights with polarization compared to TI (Table 1), which therefore indicates that if FNT contributes, it does not affect the conclusion in our manuscript.

|  | *Φ*_W-up_ (eV) | *Φ*_W-down_ (eV) | *Φ*_Au-up_ (eV) | *Φ*_Au-down_ (eV) |
| --- | --- | --- | --- | --- |
| TI | 0.85 | 0.77 | 0.78 | 0.85 |
| FNT | 1.12 | 0.83 | 0.87 | 1.14 |

Table 1: Barrier heights from TI and FNT fits

**4, Possible space-charge effect to explain unusual variation of the barrier height for LSMO/BTO/Au and LSMO/BTO/Cu junctions**

Our space-charge model succeeds to interpret the unusual data obtained in the studied PVDF-based MFM structure (main manuscript). Such space-charge effect remains also valid whatever the nature of the ferroelectric (organic or inorganic, with n- or p-type doping). For example, similar and unusual variations of barrier heights were also reported in LSMO/BTO/Au and LSMO/BTO/Cu junctions,[^5^](#_ENREF_10) these unusual variations of barrier heights can be easily understood by the model taking space-charge effect into account.

For inorganic oxide films, an excess of oxygen vacancies (n-doping defects) resulting from the poor-oxygen atmosphere in fabrication process or surface oxygen exchange close to the top surface usually happens,^6,7^ and the defect density can be as large as ~ 10^21^-10^22^ charges/cm^3^ (ref. [8-10](#_ENREF_13)). Such oxygen vacancies are expected to play the role of space charges. Charged defects distributed uniformly at both interfaces are considered for space-charge effect and the parameters used for imperfect screening effect are based on Thomas-Fermi theory (table 2, below). Based on calculations presented in Figs. 4a and 4b (in main manuscript), it is convenient to fit the experimental data with model including both space-charge effect and imperfect-screening effect. By choosing appropriate parameters, the barrier heights for both polarization orientations for LSMO/BTO/Au and LSMO/BTO/Cu junctions are shown in Fig. S4. *Φ*_1_ is for LSMO side and *Φ*_2_ is for Au or Cu metal side. The open symbols are results from the fits and the error bars are data from Fig. 4e in ref [5](#_ENREF_10). As expected, these barrier heights determined by the model considering space-charge effect are in very good agreement with experimental data. In the fits, the space-charge effect contributes of 0.22 eV (cyan color in Fig. 4b in main manuscript) and 0.33 eV (green color in Fig. 4b in main manuscript) for the LSMO/BTO/Au junctions and LSMO/BTO/Cu junctions, respectively.

It is worth mentioning that semi-classical models used here for screening effects at metal/ferroelectric interfaces are approximations. First-principles calculations have shown that the actual screening properties at a metal/ferroelectric interface depend on the bonding and not necessarily on the bulk physical parameters of the metallic electrodes (Ref. 11 for example) and then an effective screening length related to the interface properties should be considered. Instead of considering the ratio between the Thomas-Fermi screening length and the metal dielectric constant (λ_i_/ε_i_), one could write λ_i,eff_ as the effective screening length at the interface. Then, the change of the interfacial barrier height in the Thomas-Fermi approach can be written as:

$\Delta\Phi_{ei}=\frac{e\sigma_{s}\lambda_{i,eff}}{\varepsilon_{0}}$ (3)

where σ_S_ is the screening charge density. Assuming, the screening at the interfaces is imperfect, therefore σ_S_ < P. If we consider a constant electric field in the ferroelectric and the continuity of the electric displacement and potential, we can write σ_S_ as:

$\sigma_{s}=\frac{Pd}{\varepsilon_{f}\left(\lambda_{1,eff}+\lambda_{2,eff}\right)+d}\cong P$ (if $d\gg\varepsilon_{f}\left(\lambda_{1,eff}+\lambda_{2,eff}\right)$ ) (4)

Then, Eq. (1) becomes:

$\Delta\Phi_{ei}=\frac{ePd\lambda_{i,eff}}{\varepsilon_{0}\left[\varepsilon_{f}\left(\lambda_{1,eff}+\lambda_{2,eff}\right)+d\right]}$ (5)

One can then replace the expression (in Eq. 2) in the main manuscript by the above expression (Eq. 5). Note that considering such effective screening length will only affect the specific value of the variation of barrier height due to imperfect screening mechanism and thus will result in an "offset" in the values showed in Fig. 4a (in main manuscript). Moreover, this different effective screening length will not affect the contribution of the space-charge effect (Fig. 4b in main manuscript).

|  | LSMO | Au | Cu |
| --- | --- | --- | --- |
| 𝜆 (nm) | 0.4 | 0.06 | 0.55 |
| 𝜀 (𝜀_0_) | 8 | 2 | 2 |

Table 2: Screening parameters for electrodes^1, 12-15^


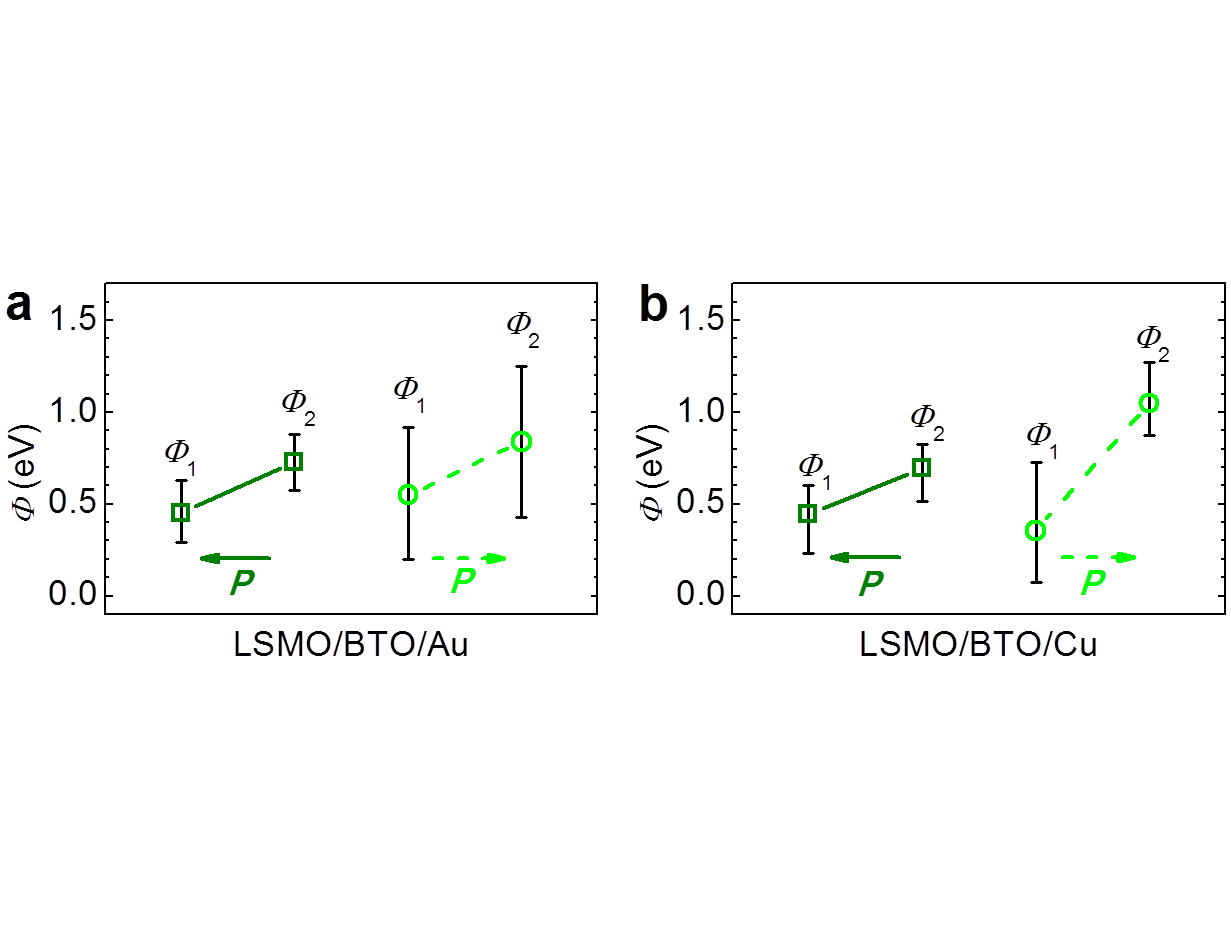


**Figure S4 |** **Barrier height fits.** Barrier heights calculated using our model including both space-charge effect and imperfect-screening effect for both polarization orientations of LSMO/BTO/Au (a) and LSMO/BTO/Cu (b) junctions. *Φ*_1_ is for LSMO side and *Φ*_2_ is for Au or Cu metal side. Open symbols are results from the fits and error bars are data from Fig. 4e in ref [5](#_ENREF_10).

**References:**

1. Pantel, D. & Alexe, M. Electroresistance effects in ferroelectric tunnel barriers. *Phys. Rev. B* **82**, 134105 (2010).

2. Kao, K.-C. & Hwang, W. Electrical transport in solids: with particular reference to organic semiconductors. (Taylor & Francis, 1979).

3. Das, R.R., Bhattacharya, P., Perez, W., Katiyar, R.S. & Bhalla, A. Leakage current characteristics of laser-ablated SrBi_2_Nb_2_O_9_ thin films. *Appl. Phys. Lett.* **81**, 880-882 (2002).

4. Fowler, R.H. & Nordheim, L. in Proceedings of the Royal Society of London A: Mathematical, Physical and Engineering Sciences, Vol. 119 173-181 (The Royal Society, 1928).

5. Soni, R. et al. Giant electrode effect on tunnelling electroresistance in ferroelectric tunnel junctions. *Nature Commun.* **5**, 5414 (2014).

6. Farokhipoor, S. & Noheda, B. Screening effects in ferroelectric resistive switching of BiFeO_3_ thin films. *APL Materials* **2**, 056102 (2014).

7. Hiboux, S. & Muralt, P. Origin of voltage offset and built-in polarization in in-situ sputter deposited pzt thin films. *Integr. Ferroelectr.* **36**, 83-92 (2001).

8. Pintilie, L. et al. Metal-ferroelectric-metal structures with Schottky contacts. II. Analysis of the experimental current-voltage and capacitance-voltage characteristics of Pb(Zr,Ti)O_3_ thin films. *J. Appl. Phys.* **98**, 124104 (2005).

9. Heywang, W. Resistivity anomaly in doped barium titanate. *J. Am. Ceram. Soc.* **47**, 484-490 (1964).

10. Heywang, W. Semiconducting barium titanate. *J. Mater. Sci.* **6**, 1214-1224 (1971).

11. Stengel, M., Vanderbilt, D. & Spaldin, N.A. Enhancement of ferroelectricity at metal-oxide interfaces. *Nature Mater.* **8**, 392-397 (2009).

12. Ku, H. & Ullman, F. Capacitance of thin dielectric structures. *J. Appl. Phys.* **35**, 265-267 (1964).

13. Gajek, M. et al. Tunnel junctions with multiferroic barriers. *Nature Mater.* **6**, 296-302 (2007).

14. Hikita, Y., Kawamura, M., Bell, C. & Hwang, H. Electric field penetration in Au/Nb: SrTiO_3_ Schottky junctions probed by bias-dependent internal photoemission. *Appl. Phys. Lett.* **98**, 192103 (2011).

15. Kim, D. et al. Polarization relaxation induced by a depolarization field in ultrathin ferroelectric BaTiO_3_ capacitors. *Phys. Rev. Lett.* **95**, 237602 (2005).
